# Differentially expressed microRNAs in experimental cerebral malaria and their involvement in endocytosis, adherens junctions, FoxO and TGF-β signalling pathways

**DOI:** 10.1038/s41598-018-29721-y

**Published:** 2018-07-26

**Authors:** Aarón Martin-Alonso, Amy Cohen, María Antonieta Quispe-Ricalde, Pilar Foronda, Agustín Benito, Pedro Berzosa, Basilio Valladares, Georges E. Grau

**Affiliations:** 1 Instituto Universitario de Enfermedades Tropicales y Salud Pública de Canarias, Universidad de La Laguna, La Laguna, Islas Canarias Spain; 2 Vascular Immunology Unit, Department of Pathology, The University of Sidney, Sydney, Australia; 3 Department of Biology, Faculty of Sciences, National University of San Antonio Abad of Cusco, Cusco, Peru; 4 National Centre for Tropical Medicine, Health Institute Carlos III (ISCIII in Spanish), Madrid, Spain; 5 Network Biomedical Research on Tropical Diseases (RICET in Spanish), Madrid, Spain

## Abstract

Cerebral malaria (CM) is the most severe manifestation of infection with *Plasmodium*, however its pathogenesis is still not completely understood. microRNA (miRNA) have been an area of focus in infectious disease research, due to their ability to affect normal biological processes, and have been shown to play roles in various viral, bacterial and parasitic infections, including malaria. The expression of miRNA was studied following infection of CBA mice with either *Plasmodium berghei* ANKA (causing CM), or *Plasmodium yoelii* (causing severe but non-cerebral malaria (NCM)). Using microarray analysis, miRNA expression was compared in the brains of non-infected (NI), NCM and CM mice. Six miRNA were significantly dysregulated between NCM and CM mice, and four of these, miR-19a-3p, miR-19b-3p, miR-142-3p and miR-223-3p, were further validated by qPCR assays. These miRNA are significantly involved in several pathways relevant to CM, including the TGF-β and endocytosis pathways. Dysregulation of these miRNA during CM specifically compared with NCM suggests that these miRNA, through their regulation of downstream targets, may be vitally involved in the neurological syndrome. Our data implies that, at least in the mouse model, miRNA may play a regulatory role in CM pathogenesis.

## Introduction

Malaria is a serious and sometimes life-threatening disease transmitted by the bite of female *Anopheles* mosquitoes. In 2015 alone, the disease led to an estimated 429,000 deaths, with sub-Saharan Africa the most heavily affected with 90% of this count. Malaria is most severe in children under five years old, who account for 70% of all death^1^. Features of the life-threatening disease typically consist of metabolic acidosis, severe anaemia, and cerebral malaria (CM), with the latter eventuating in approximately 1% of cases worldwide, equal to 1 million people^1^. CM is an often fatal complication of malaria, and the most severe manifestation of the infection, characterised by unarousable coma and unconsciousness, often leading to death, or the occurrence of neurological sequelae in survivors^2^. Currently the only treatment options for CM are a combination of immediate intensive care and anti-malarial medications^3^, however resistance is increasingly common, and access to adequate medical care in time is not. Non-cerebral malaria (NCM) is another manifestation of severe malaria, resulting in severe anaemia and respiratory distress, and can be caused by other strains of *Plasmodium*, including *P*. *vivax*^4^.

Of particular pertinence to this study, the pathology of CM is not currently completely understood, though several proposed hypotheses have been proposed. The pathogenicity associated with CM is understood to involve parasitised red blood cells (PRBCs), endothelial cells, various adhesion molecules and cytokines, platelets, monocytes, and microvesicles released from many different cell types, including several involved in the pathogenesis of CM^5–8^. Within brain microvessels, and in response to the parasite itself, sequestration occurs – that is, the cytoadherence of PRBCs, platelets, and monocytes to endothelial and intravascular cells^5,6,8^. This accumulation of cells within the vessels, mediated by adhesion markers such as ICAM-1, VCAM-1, and E-selectin, can trigger changes in the function and viability of the cerebral endothelium, destruction of microcirculatory blood flow, and hypoxia, leading to blood brain barrier dysfunction and, consequently, several of the clinical manifestations observed in CM^5,7,8^. The loss of blood-brain barrier integrity also results in increased permeability, leading to increased cerebral oedema, as observed in patients^9,10^. Parasite infection, as stated previously, induces the overproduction of cytokines, such as TNF, lymphotoxin, and IFN-ɣ, and this also results in alterations of the cerebral endothelium, upregulation of adhesion molecules, EC apoptosis, and further mediation of cytokine and chemokine release^11^. In recent times, microRNA (miRNA) have also been investigated as to their role in normal conditions and deregulation in a number of diseases, including malaria.

miRNA are small, single-stranded non-coding RNA that have been widely demonstrated to be able to regulate the expression of at least one third of the human genome and play a critical role in a variety of normal biological processes, including cell differentiation, apoptosis, development, and metabolism^12^. Changes in miRNA expression have also been identified in a range of diseases, including viral, bacterial, and parasitic infections^13–15^, including malaria. miRNA expression changes in the liver and brain have been studied during malaria using the murine model^16–18^, and within circulating microvesicles^19,20^, investigating the effect of *Plasmodium* infection on miRNA signatures within these tissues and cells. They found that certain miRNA were down or upregulated, including miR-16, miR-27a, miR-150, and miR-451, in response to infection, and that blocking some of these protects mice against the development of the infection. The effect of anti-malarial treatment on this expression has also been tested, with miRNA expression changes being reversed in some cases^21–23^. These studies provide supporting evidence of the roles that miRNA can play in the pathogenesis of, resistance to, and protective immune response against malarial infection.

However, the exact mechanism of action of miRNA during infection, and whether they play either a pathogenic or a protective role, or a combination of the two, is still not completely understood. Therefore, there is still a need for further research in this area. Our study examines the role of miRNA within brain tissue during experimental cerebral or non-cerebral malaria, and in non-infected mice in comparison. We show that, after infection, there are changes in specific miRNA in the brain tissue which could provide prospective avenues of research into further understanding CM pathogenesis and how to treat or prevent it.

## Materials and Methods

### Mice

All mice were handled according to approved protocols of the University of Sydney Animal Ethics Committee (protocol number 2015/832). 7-10-week-old female CBA mice (Animal Resource Centre, Perth) were housed under pathogen-free conditions, fed a commercial rodent pellet diet, and had access to water *ad libitum*. For experimental comparisons, three groups of five mice were included: non**-**infected (NI), *Plasmodium berghei* ANKA-infected (PbA, CM), and *Plasmodium yoelii*-infected (Py, NCM). PbA infection of CBA mice leads to fatal disease with cerebral pathology during the neurological phase, between day 6 and day 14 post-infection (p.i.)^24^. Mice surviving beyond this point are defined as NCM: i.e. Py infection leads to a fatal but non-cerebral syndrome due to hyperparasitaemia and severe anaemia, 14 or more days post infection.

### *Plasmodium* inoculation

All infections were initiated by intraperitoneal injection of 1 × 10^6^ PbA/Py PRBCs, as previously described^25^. Parasitaemia was monitored by counting PRBCs in Diff-Quick-stained thin blood smears on day four p.i. and every 1–2 days after this, for the duration of the infection. Mice were assessed using a clinical evaluation score, and CM diagnosed if mice presented with ruffled fur, severe motor impairment (ataxia, hemiplegia, or paraplegia) or convulsions and was given a score of 3 or 4 as described previously^26^. This diagnosis was then confirmed by histopathology, i.e. presence of leucocyte and PRBC adherence to endothelium, haemorrhages, and oedema surrounding vessels. Py-infected mice without any of the symptoms mentioned above were classified as NCM.

### Brain tissue preparation

At the time of CM for PbA-infected mice, all groups of mice were sacrificed as detailed above and brain tissue was collected from infected (CM and NCM), and NI mice. Samples were placed immediately in RNAlater (Life Technologies) in a 1:5 volume ratio for complete submersion.

### RNA extraction

Total RNA including miRNA was isolated using Ambion mirVana^TM^ miRNA Isolation Kit (Life Technologies). The concentration and purity of total RNA was determined using a NanoDrop-1000 spectrophotometer (only RNA samples with A260/A280 and A260/A230 ratios above 2.0 and 1.8, respectively, were included into subsequent miRNA profiling studies).

### cDNA synthesis and preamplification

100 ng of total RNA was reverse transcribed using the microRNA reverse transcription (RT) reaction kit by following manufacturer’s protocol. After reverse transcription, a pre-amplification step was performed as per the manufacturer’s recommendation in order to increase the quantity of desired cDNA before performing PCR, and to significantly increase the ability to detect low abundance transcripts. The preamplified product was diluted 40 times by mixing 4 μl of the preamplified product with 156 μl of 0.1x Tris-EDTA (TE), pH 8.0.

### OpenArray analysis

After mixing 1:1 with TaqMan OpenArray master mix, pre-amplified samples were loaded onto TaqMan OpenArray MicroRNA Panels by using the Accufil system and run on the QuantStudio 12 K Flex system (Life Technologies), as per the manufacturer’s instructions. 754 rodent miRNA were amplified in each sample, with technical replicates and internal controls. All reactions were performed in duplicate, and each technical replicate was analysed on two different OpenArray® Panels. The comparative (ΔΔ) C_T_ method was used to quantify relative expression across all miRNA and samples. Resulting Crt values were filtered to exclude low-quality reactions with an amplification score below 1.1, following recommendations of Life Technologies technicians, where Crt is defined as the PCR cycle number at which the fluorescence meets the threshold in the amplification plot. miRNA with a Crt value >29 were considered unamplified. This Crt cut-off was chosen since OpenArray® reactions are carried out in small volumes (33 nL); a single molecule is more concentrated in a smaller reaction volume and amplifies sooner than it would from regular microplate qPCRs. More concretely, single copy numbers will produce, on average, a Crt value of 29 with TaqMan® assays^27^. Furthermore, for large scale miRNA expression profiling studies, it has been demonstrated that global normalisation outperforms the normalisation strategy that makes use of small RNA controls^28^. Consequently, raw Crt values were normalised using global normalisation of each pool separately, as per recommendations. After global normalisation, technical replicates differing by more than 0.5 standard deviations (SD) were excluded from the analysis, and average ΔCt values were estimated for the remaining results. Finally, miRNA which were not amplified in more than 50% of samples were considered to be lowly expressed and, therefore, also excluded from the analysis. Using these criteria, 395 miRNA were filtered out leaving 359 miRNA in the final data analysis.

### RT-qPCR analysis

The abundance of those miRNAs that showed a differential expression between the NCM and the CM group with the OpenArray platform was further validated by TaqMan MicroRNA assays, following the manufacturer’s instructions. To this end, five NCM four CM brain samples were included in this analysis. The starting RNA concentration was set at 10 ng and each sample was tested in triplicate. U6 and snoRNA-202 were identified as suitable endogenous controls and used in this validation step. Real time qPCR was carried out on a StepOne Real Time PCR System. Technical replicates for which the SD was >0.5 were removed.

### Pathway analysis

DIANA-mirPath v3.0 was used for analysing the combinatorial effect of dysregulated miRNAs of interest on target pathways, with a False Discovery Rate (FDR) of 5%. DIANA mirPath is a powerful tool to analyse the combinational effects of miRNA on signalling pathways^29^. This bioinformatics software performs an enrichment analysis of the input datasets by comparing the set of dysregulated miRNA to all available biological pathways provided by the Kyoto Encyclopedia of Genes and Genomes (KEGG)^30^. It should be taken into account that, to this end, we used TarBase^31^ that houses a manually curated collection of experimentally proven miRNA targets. Classic miRNA target prediction algorithms were not used due to their intrinsic high false positive rates^32^. The combinatorial effect of co-expressed miRNA in the modulation of a given pathway is taken into account by the simultaneous analysis of multiple miRNA. The significantly influenced pathways (p < 0.05) were identified.

### Statistical analyses

With regards to OpenArray results, differences between experimental groups were assessed by Kruskal-Wallis with Dunn post-hoc test, and p-values were adjusted for multiple testing using the Benjamini-Hochberg method. The results were calculated using the ΔΔCt method^33^, and fold change (FC) was calculated using the 2^−ΔΔCt^ method, and then data were log2-transformed. Those miRNA with expression differences among the three study groups (as determined by Kruskal-Wallis test and corresponding post-hoc tests) were included in hierarchical clustering, which was performed using Euclidean distance, complete linkage and z-score normalisation. On the other hand, the non-parametric Mann-Whitney U test was used to compare the level of expression between the NCM and CM group with qPCR validation results. P-values of <0.05 (two-sided) were considered significant. All data were analysed using SPSS Statistics 20 (IBM Corporation), GraphPad Prism version 5.02 (GraphPad Software) and RStudio.

The datasets generated and analysed during the current study are available from the corresponding author on reasonable request.

## Results

**Figure 1:**
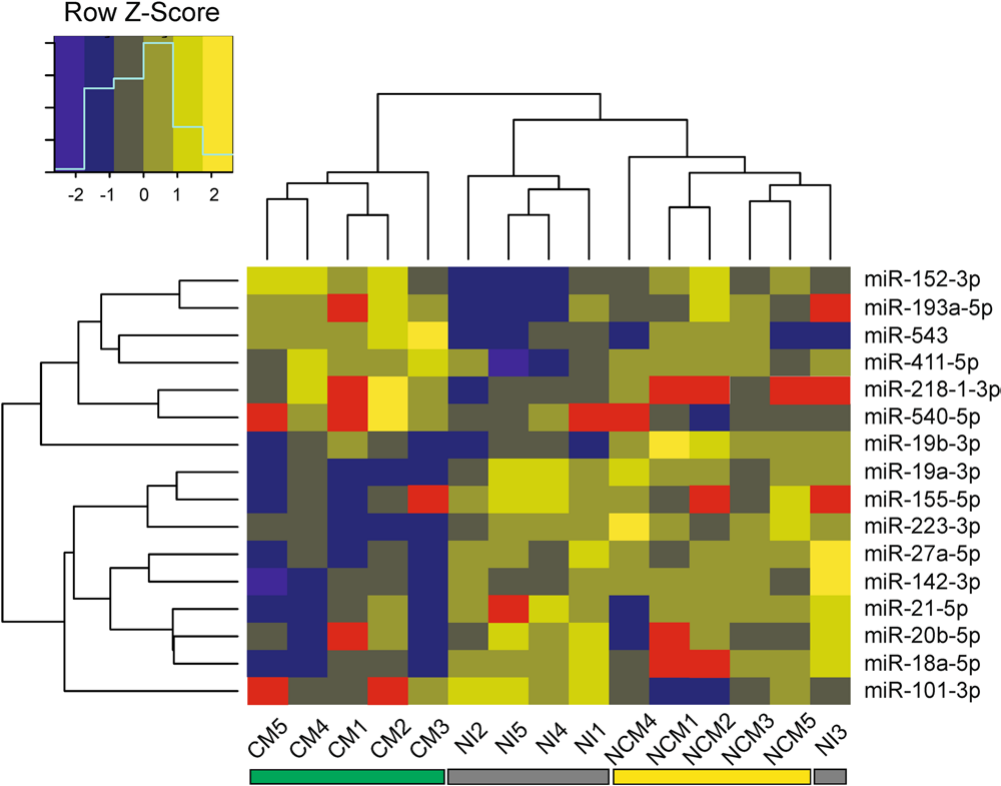
Hierarchical clustering of dysregulated miRNA based on Euclidean distance measure and complete linkage with Z score normalised gene expression. Each row represents one miRNA and each column represents one sample. Red rectangles represent missing values. NI: non-infected; NCM: *Plasmodium yoelii*-infected mice; CM: *Plasmodium berghei* ANKA-infected mice.

**Figure 2:**
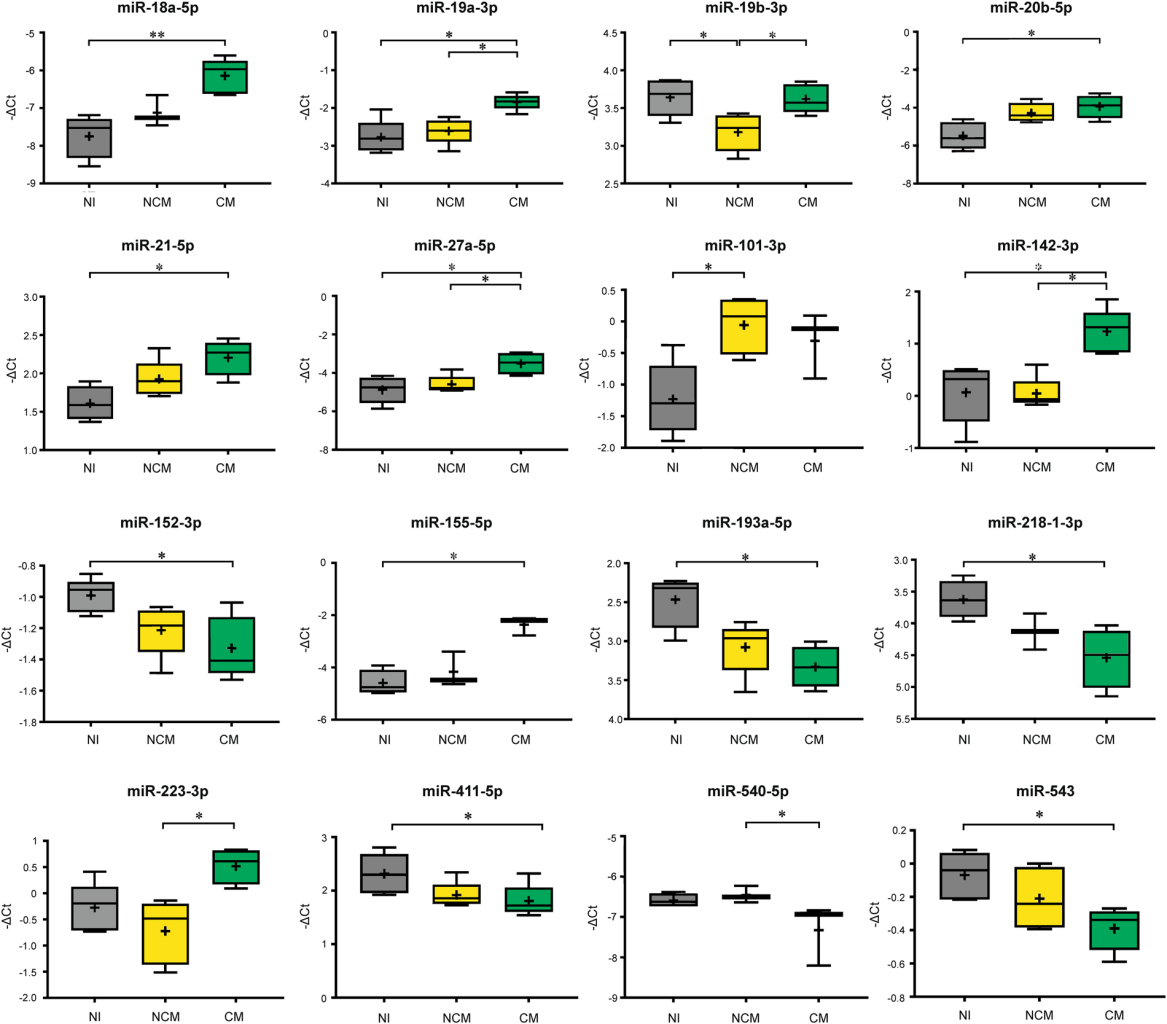
Box plots show miRNA expression levels measured in the three study groups, expressed as normalised values using global normalisation. The horizontal line denotes the median value, the box encompasses the upper and lower quartiles, whiskers show the range, and the plus symbol denotes the mean. A nonparametric Kruskal-Wallis test was carried out. If the Kruskal-Wallis test was significant, *post hoc* tests were carried out, to adjust p-values for multiple testing using the Benjamini-Hochberg method. The results of these are denoted in the plot with horizontal bars and asterisks (*P = 0.01–0.05; **P < 0.01). These data represent results of duplicate experiments, with 5 animals in each group.

The changes in miRNA expression in the brains of NI, NCM and CM mice were examined using OpenArray® analysis. Unsupervised hierarchical clustering of this set of miRNA separated the samples into two main clusters suggesting homogeneity of the miRNA expression profiles within each group. Whilst all CM samples were placed in one cluster, the other cluster was constituted by the other two experimental groups, NI and NCM mice (Fig. 1). According to this clustering method, the relative expression of dysregulated miRNA in NCM mice was more closely related to the NI group than to CM mice. Regarding the set of dysregulated miRNA, two main clusters were identified, the first one comprising those miRNA that were upregulated in CM mice whereas those downregulated were included in the second cluster. The log2 (fold change) was calculated (Table 1), and graphed in Fig. 2 together with the calculations regarding the significance described above.

**Table 1:** The list of significantly dysregulated miRNA is shown, with log_2_ fold change (log_2_FC) and significance calculated for each comparison and each miRNA.

miRNA	Kruskal-Wallis test	Log_2_FC, with Dunn’s + Benjamini-Hochberg Post-Hoc tests		
		NI vs. NCM	NI vs. CM	NCM vs. CM
miR-18a-5p	<0.01		1.61	
miR-19a-3p	<0.05		0.93	0.77
miR-19b-3p	<0.05	−0.46		0.44
miR-20b-5p	<0.05		1.55	
miR-21-5p	<0.05		0.60	
miR-27a-5p	<0.05		1.37	1.09
miR-101-3p	<0.05	1.17		
miR-142-3p	<0.01		1.17	1.19
miR-152-3p	<0.05		−0.34	
miR-155-5p	<0.05		2.24	
miR-193a-5p	<0.05		−0.87	
miR-218-1-3p	<0.05		−0.92	
miR-223-3p	<0.05			1.44
miR-411-5p	<0.05		−0.51	
miR-540-5p	<0.05			−0.87
miR-543	<0.05		−0.32

We found twelve miRNA to be significantly dysregulated between NI and CM mice: miR-18a-5p, miR-19a-3p, miR-20b-5p, miR-21-5p, miR-27a-5p, miR-142-3p, miR-152-3p, miR-155-5p, miR-193a-5p, miR-218-1-3p, miR-411-5p, and miR-543 (Fig. 2). Two miRNA were differentially expressed between NI mice and NCM mice: miR-19b-3p and miR-101-3p; and six miRNA between NCM and CM mice: miR-19a-3p, miR-19b-3p, miR-27a-5p, miR-142-3p, miR-223-3p, and mmu-miR-540-5p. Three miRNA: miR-19a-3p, miR-27a-5p, and miR-142-3p were significantly upregulated in CM mice compared with NI as well as NCM, with NCM being an intermediate in this expression comparison.

Of those miRNA significantly dysregulated in CM vs. NI mice, miR-18a-5p, miR-19a-3p, miR-20b-5p, miR-21-5p, miR-27a-5p, miR-142-3p, and miR-155-5p were increased 0.60-2.24 log2-fold, and miR-152-3p, miR-193a-5p, miR-218-1-3p, miR-411-5p, and miR-543 were decreased 0.32–0.92 log2-fold. miR-101-3p was increased 1.17 log2-fold, and miR-19b-3p was decreased 0.46 log2-fold in NCM vs. NI mice. Finally, in comparing NCM vs. CM mice, miR-19a-3p, miR-19b-3p, miR-27a-5p, miR-142-3p, and miR223-3p were increased 0.44–1.44 log2-fold, and miR-540-5p was decreased 0.87 log2-fold in CM mice.

**Figure 3:**
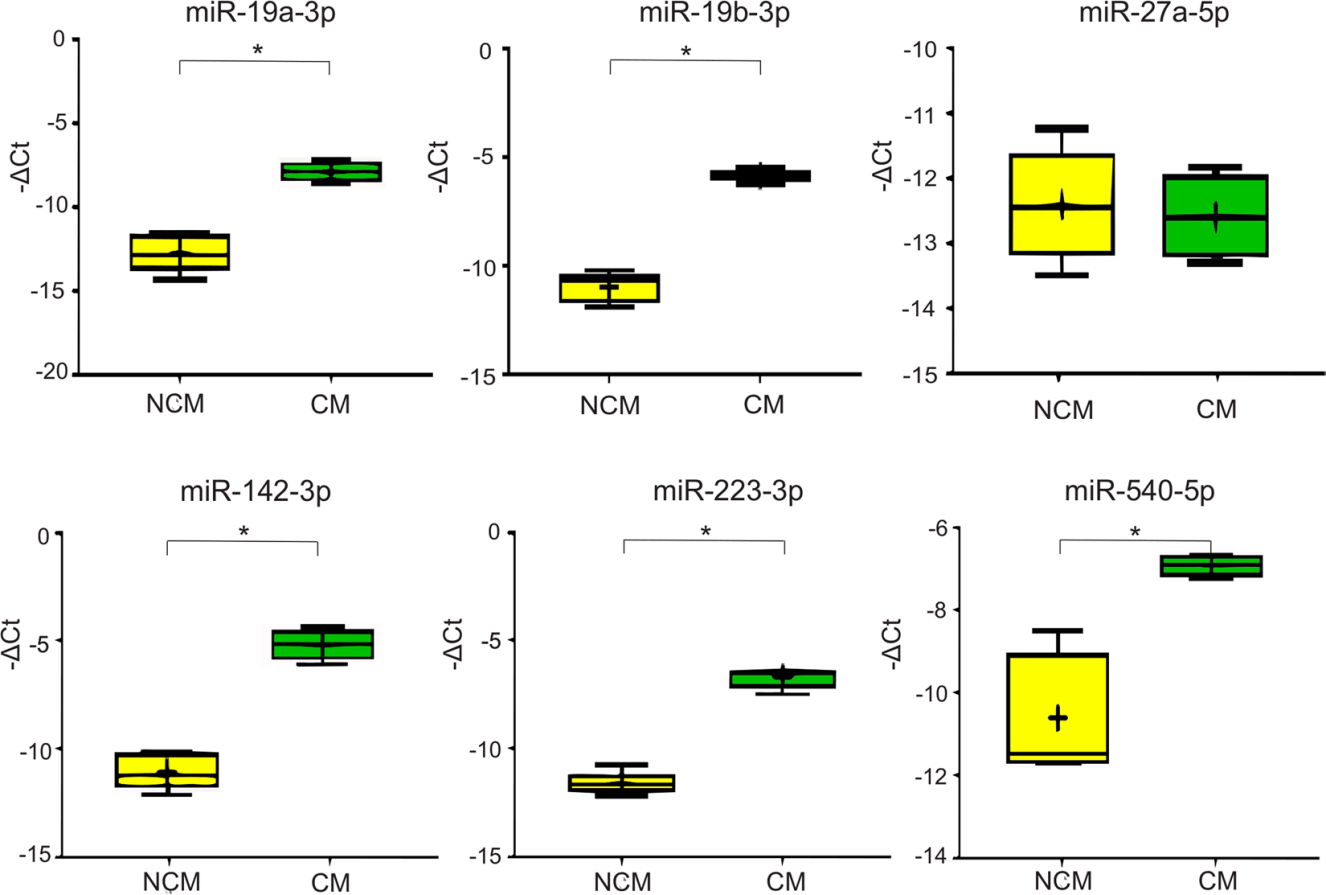
Boxplots showing the qPCR validation results. The expression levels of miR-19a-3p, miR-19b-3p, miR-27a-5p, miR-142-3p, miR-223-3p and miR-540-5p, selected according the OpenArray results outcome, were measured by real-time RT-PCR. The horizontal line denotes the median value, the box encompasses the upper and lower quartiles, whiskers show the range, and the plus symbol denotes the mean. Mann-Whitney test results are denoted in the plot with horizontal bars and asterisks (*P < 0.05).

Our previously identified miRNA of interest – that is, the six miRNA shown to be differentially expressed between NCM and CM mouse brain tissue by OpenArray analysis, were analysed for validation by RT-qPCR. Significant changes were found in the expression of miR-19a-3p, miR-19b-3p, miR-142-3p, and miR-223-3p, as shown in Table 2 and Fig. 3, verifying the differences in miRNA abundance in CM compared with NCM found in OpenArray analysis. A significant change of expression was determined for miR-540-5p in NCM compared with CM mice, however this was the opposite change in abundance to that found by OpenArray analysis. No significant difference was found in the abundance of miR-27a-5p between NCM and CM conditions by RT-qPCR. Hierarchical clustering was repeated including only this set of six miRNA and qPCR results, and the samples belonging to each biological group were clustered separately as observed with OpenArray results (data not shown).

**Table 2:** Comparison of results obtained by RT-qPCR and OpenArray analysis.

miRNA	Significance of fold change in CM vs. NCM					
	OpenArray			RT-qPCR		
	Log2 fold change	p-value	Up- or downregulated	Log2 fold change	p-value	Up- or downregulated
miR-19a-3p	0.77	<0.05	Up	4.86	<0.05	Up
miR-19b-3p	0.44	<0.05	Up	5.24	<0.05	Up
miR-27a-5p	1.09	<0.05	Up	0.1	n.s.	—
miR-142-3p	1.19	<0.05	Up	5.98	<0.05	Up
miR-223-3p	1.44	<0.05	Up	4.9	<0.05	Up
miR-540-5p	−0.87	<0.05	Down	3.73	<0.05	Up

We then performed pathway analysis on miRNA differentially expressed between CM and NCM, in order to identify miRNA with potential roles specifically in the cerebral syndrome. Those miRNA that were upregulated in CM mice in comparison with NCM mice as determined by qPCR validation analyses (miR-19a-3p, miR-19b-3p, miR-142-3p, miR-223-3p and miR-540-5p) were analysed using the DIANA-mirPath v3.0 software, which identified 16 pathways in mice as significantly enriched (p < 0.05) (Table 2). The pathway relating to endocytosis (3^rd^ in the list, p = 2.04E^−5^), as well as the FoxO (6^th^, p = 0.0005) and TGF-β signalling (10th, p = 0.007), and adherens junctions (13^th^, p = 0.01) pathways were among the most significantly regulated by our list of miRNA of interest. This is particularly interesting as the endocytosis, FoxO signalling and adherens junctions pathways are all intrinsically linked to the TGF-β signalling pathway, as they share common elements. Two of these miRNA that were more abundant in CM mice (miR-19a-3p and miR-19b-3p) target at least 28 of the genes involved in the FoxO signalling pathway, 16 of the genes participating in the TGF-β signalling pathway, and 20 of the genes participating in the adherens junctions pathway.

When analysing the combinatorial effect of those miRNA upregulated in CM mice (when compared to NCM mice) to target pathways in humans, 34 pathways were identified by the software as significantly enriched (p < 0.05), including 11 of those also significantly enriched in mice. Of these, the FoxO (4^th^, p = 5.28E^−7^) and TGF-β signalling (32^th^, p = 0.023) remained as significantly enriched as identified in mice, although the number of experimentally proven targets of these molecules of interest is generally higher in humans than in mice in the TarBase database. Specifically when analysing target pathways in humans, three of the miRNA upregulated in CM mice (miR-19a-3p, miR-19b-3p and miR-223-3p) target 43 of the genes involved in the FoxO signalling pathway, whilst miR-19a-3p and miR-19b-3p regulate the expression of 16 of the genes participating in the TGF-β signalling pathway.

## Discussion

This study provides the first large-scale analysis of miRNA expression in experimental malaria and reveals that *Plasmodium* infection induces the dysregulation of a specific set of miRNA in the brain, among which are several miRNA that are more abundant in CM compared with NCM mice. This group of miRNA likely plays important roles in CM, and includes several molecules that are widely associated with inflammatory responses. For instance, miR-223 was found to be overexpressed in rheumatoid arthritis^34^, osteoarthritis^35^, and inflammatory bowel disease^36^, among others. Furthermore, miR-223 actively participates in infection processes, regulating the differentiation of several key players of the innate immune response and therefore, likely plays an important role in the early stages of infection^37^.

Clustering analyses of the OpenArray and qPCR results suggested that those miRNA that showed a differential expression may represent a miRNA fingerprint of CM, therefore able to distinguish between individuals with and without cerebral complications during *Plasmodium* infections. Nevertheless, the levels of expression of this set of miRNA should be further analysed in blood samples from patients with CM and compared to NCM patients, in order to confirm their usefulness as biomarkers of CM in humans. This will allow us to characterise the cellular source of these miRNA further, and quantify how the differences in immune cell influx into the brain during different disease phases affects miRNA expression, in the future. The utility of miRNA as biomarkers of infection has been widely demonstrated in several parasitoses, including schistosomiasis^38^, filarial infections^39^, and toxoplasmosis^40^. Among the set of miRNA dysregulated in CM mice, miR-223 has been defined as a potential biomarker of infection with Schistosoma japonicum^38^.

Regarding the relationship between miRNA and malaria, a recent study has demonstrated that intrinsic miRNA levels play a significant role in malaria resistance^41^, proving that miRNA activity may be utilised to defend host cells against complex eukaryotic pathogens, not only against viruses as previously demonstrated^42^. LaMonte *et al*. showed that miR-223, among others, strongly reduces the growth of *P*. *falciparum* in erythrocytes carrying a variant haemoglobin allele (HbS), which causes sickle cell disease. The authors indicated that the antimalarial activity of erythrocyte miRNA requires their entry and subsequent modification of parasite mRNA. Consequently, it is possible that the upregulation of at least miR-223 in CM mice may be a targeted anti-parasitic immune response, and that miRNA could be used as valuable targets for new therapeutic interventions in malaria – a method already being explored in other diseases.

RT-qPCR analysis was employed to validate the expression of six miRNA significantly dysregulated in CM conditions, omitting the preamplification step previously employed in the OpenArray analysis. The preamplification step, which aims to uniformly increase the levels of miRNAs in the sample, may introduce a bias, therefore validation is necessary, as previously demonstrated^43–45^. Through RT-qPCR analysis, four miRNA were validated as more abundant in CM conditions compared with NCM: miR-19a-3p, miR-19b-3p, miR-142-3p and miR-223-3p, and these were further investigated by pathway analysis. As a result, we found that some of these miRNA molecules are involved in regulating several signalling pathways in CM. Bioinformatics tools indicated that hsa-miR-19a-3p, which was upregulated in CM mice compared to NCM mice, can regulate the expression of at least 14 out of the 38 genes involved in the TGF-β signalling pathway, as well as many other genes involved in other significantly associated pathways.

TGF-β is a superfamily of cytokines, known to regulate cell differentiation, proliferation, apoptosis and pro- and anti-inflammatory immune responses^46^. TGF-β is considered a pivotal regulator of the mammalian response to malaria parasite infection, having been described as maintaining “immunological balance” during infection^47^. In our study, we found that two miRNA upregulated in CM compared with NCM mice have been experimentally demonstrated to target 16 genes from the TGF-β signalling pathway, which could lead to a miRNA-mediated downregulation of their corresponding mRNAs, subsequently altering this pathway, as is consistent with previous findings (more detailed information in Fig. S1 and Table S1)^48^. Similarly, low levels of TGF-β1 have been shown to contribute to increased disease severity in mice^49^ and humans^50^. Therefore, we suggest that the paramount relevance of the TGF-β signalling pathway to *Plasmodium* infections is, at least partly, due to the action of these miRNA.

The most significant relationship was found between miRNA upregulated in CM mice and the endocytosis pathway. Endocytosis is a mechanism for cells to remove nutrients, plasma membrane proteins, and lipids from the cell surface into the cell interior, by phagocytic or pinocytic methods^51^. This relationship is of particular interest due to the known role of microvesicles (submicron vesicles released from cell types involved in CM), where a significant overproduction is observed in cerebral infection cases only, and not in NI or NCM conditions^52^. Microvesicles have their effect on target tissues through the process of endocytosis into these target cells, therefore it is poignant that this pathway should be the most significantly regulated by our miRNA of interest. This link supports existing evidence of the role of microvesicles in CM in effecting change within the brain in response to infection^11^. Particularly, recognition of the expression of phosphatidylserine on the surface of microvesicles is crucial for phagocytic endocytosis and the subsequent clearance of microvesicles, providing evidence of a potential protective role played by miRNA in the brain.

The KEGG pathway analysis also showed relationship between miRNA upregulated in CM mice and the FoxO signalling pathway. The FoxO family of transcription factors is central to the integration of growth factor signalling, oxidative stress and inflammation^53^, by regulating the expression of several antioxidant enzyme genes, including superoxide dismutase (sod), and catalase (cat)^54^. Recently, it was shown that these antioxidant enzyme genes were altered at the transcriptional level during *Plasmodium* infection, indicated by the gradually diminished expression of superoxide dismutase-1 (sod-1), sod-2, sod-3 and catalase genes^55^. This family of transcription factors also regulates the expression of genes in cellular physiological events including apoptosis and glucose metabolism and, considering that impaired glucose production caused by the inhibition of gluconeogenesis is a frequently encountered complication in *falciparum* malaria^56^, the possible relationship among miRNA, the FoxO signalling pathway and glucose concentration should be addressed in the future.

This set of miRNA is involved in the assembly/disassembly of adherens junctions which, together with tight junctions, ensure the integrity of vascular endothelial cells forming a barrier that plays a crucial role in the health and integrity of the blood-brain barrier (BBB). Disruption of the BBB is a hallmark of immune mediated neurological disorders, including CM^57^. More concretely, during *Plasmodium* infection, the endothelium becomes activated leading to leakage associated with the disruption of endothelial cell junctional proteins^58^. However, the relationship of miRNA with the disruption of the BBB is almost unknown, but may be linked to the Vascular Endothelial cadherin (VE-cad), a component of the adherens junction complex that has emerged as an important regulator of endothelial cell-cell adhesion^59^. It has been shown that, in the presence of high levels of miR-142-3p (also upregulated in CM mice in our study), VE-cad (cdh5) levels are suppressed, decreased levels of which are known to compromise endothelial cell-cell adhesion thus leading to loss of vascular integrity and remodelling^60^. Furthermore, another component of adherens junctions, β-catenin, has also been found to be targeted in humans by miR-142-3p. Strikingly, β-catenin has also been shown to regulate FoxO function under conditions of oxidative stress^61^, indicating cross-talk between both pathways.

It is clear that the differences in expression of these miRNA during CM specifically compared with NCM suggests that they, through their regulation of downstream targets, may be vitally involved in the neurological syndrome^62^, although this statement must be further confirmed in future studies by analysing the effect of immune cells sequestered to the brain on the miRNA abundance. Collectively, these findings may help to decipher which and how miRNA are connected to some of the processes that have been historically implicated in CM pathogenesis, such as excessive pro-inflammatory cytokine production^63,64^, loss of endothelial barrier function^65,66^, and endothelial dysregulation^67^. Nevertheless, how our miRNA of interest are regulating these pathways should be analysed more thoroughly in the future due to the complexity of the interactions of these miRNA with their numerous mRNAs targets within these pathways.

## Electronic supplementary material

Supplementary Information
