# Supplementary material for: Differentially expressed microRNAs in experimental cerebral malaria and their involvement in endocytosis, adherens junctions, FoxO and TGF-β signalling pathways
Source: Sci Rep. 2018 Jul 26;8:11277. doi: 10.1038/s41598-018-29721-y (PMC6062515; doi:10.1038/s41598-018-29721-y)
Supplement: Supplementary file 1 — Supplementary Information [file 41598_2018_29721_MOESM1_ESM.pdf]

Title: Differentially expressed microRNAs in experimental cerebral malaria and their involvement in endocytosis, adherens junctions, FoxO and TGF-β signalling pathways

Authors: Aarón Martin-Alonso[1, +, *], Amy Cohen[2, +], María Antonieta Quispe-Ricalde[3], Pilar Foronda[1], Agustín Benito[4], Pedro Berzosa[4], Basilio Valladares[1], Georges E. Grau[2].

1 University Institute of Tropical Diseases and Public Health of the Canary Islands, University of La Laguna, La Laguna, Islas Canarias, España.
2 Sydney University, Vascular Immunology Unit, Department of Pathology, Sydney, Australia
3 Department of Biology, Faculty of Sciences, National University of San Antonio Abad of Cusco, Cusco, Peru
4 National Centre for Tropical Medicine, Health Institute Carlos III (ISCIII in Spanish), Madrid, Spain, Network Biomedical Research on Tropical Diseases (RICET in Spanish), Madrid, Spain
+ shared first authors

Figure S1-4. DIANA mirPath analysis of five upregulated miRNA in CM mice. Genes targeted by more than one miRNA are shown in orange boxes whereas genes targeted by one miRNA are highlighted in yellow boxes. Numbers in italics represent that several genes whose protein products play a similar role are targeted by these upregulated miRNA, detailed below.

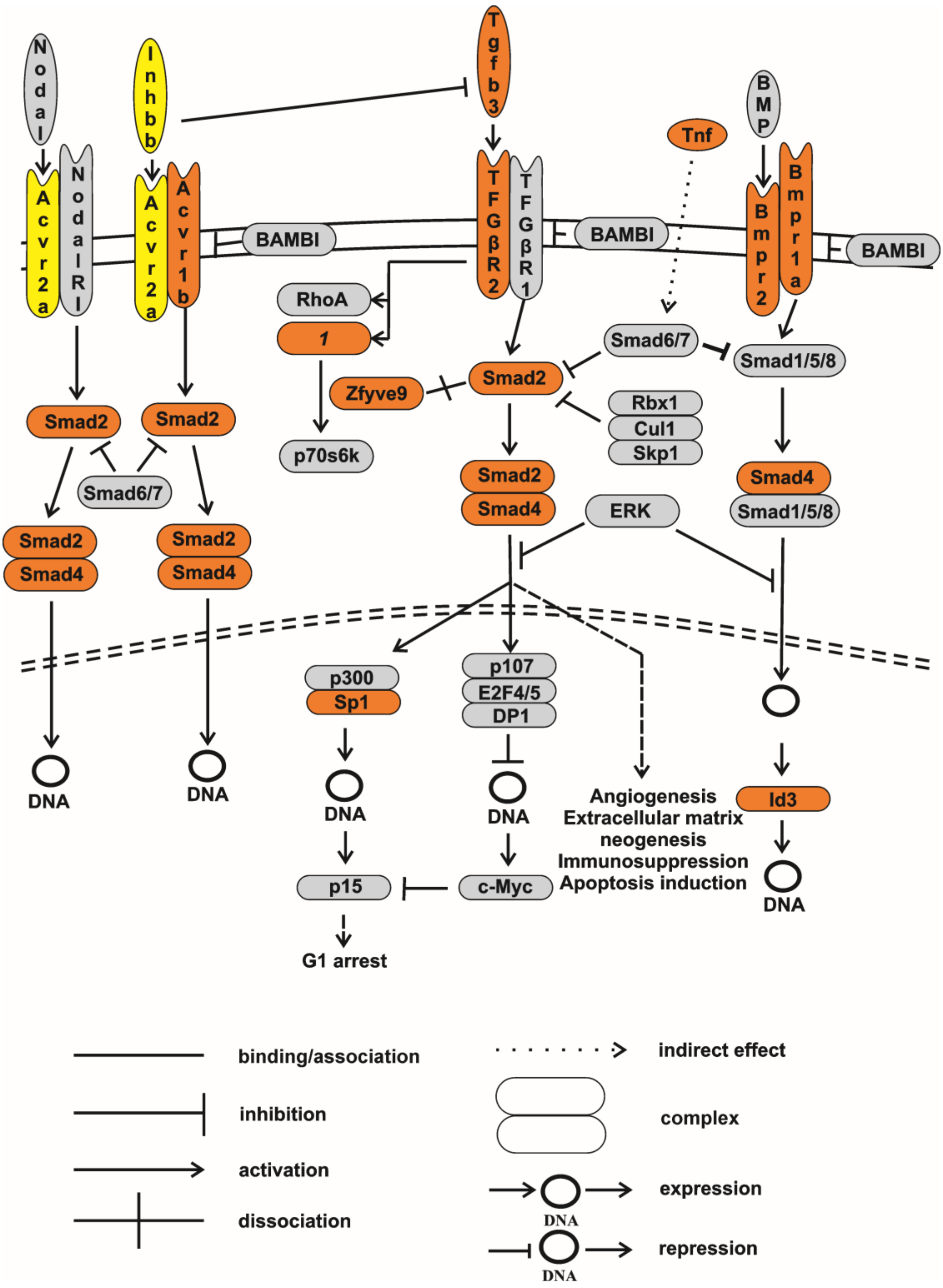

Figure S1: DIANA mirPath analysis predicted that 16 genes involved in TGF-β signalling pathway are targeted by two miRNA from this group. *1*: Ppp2ca and Ppp2r1b.

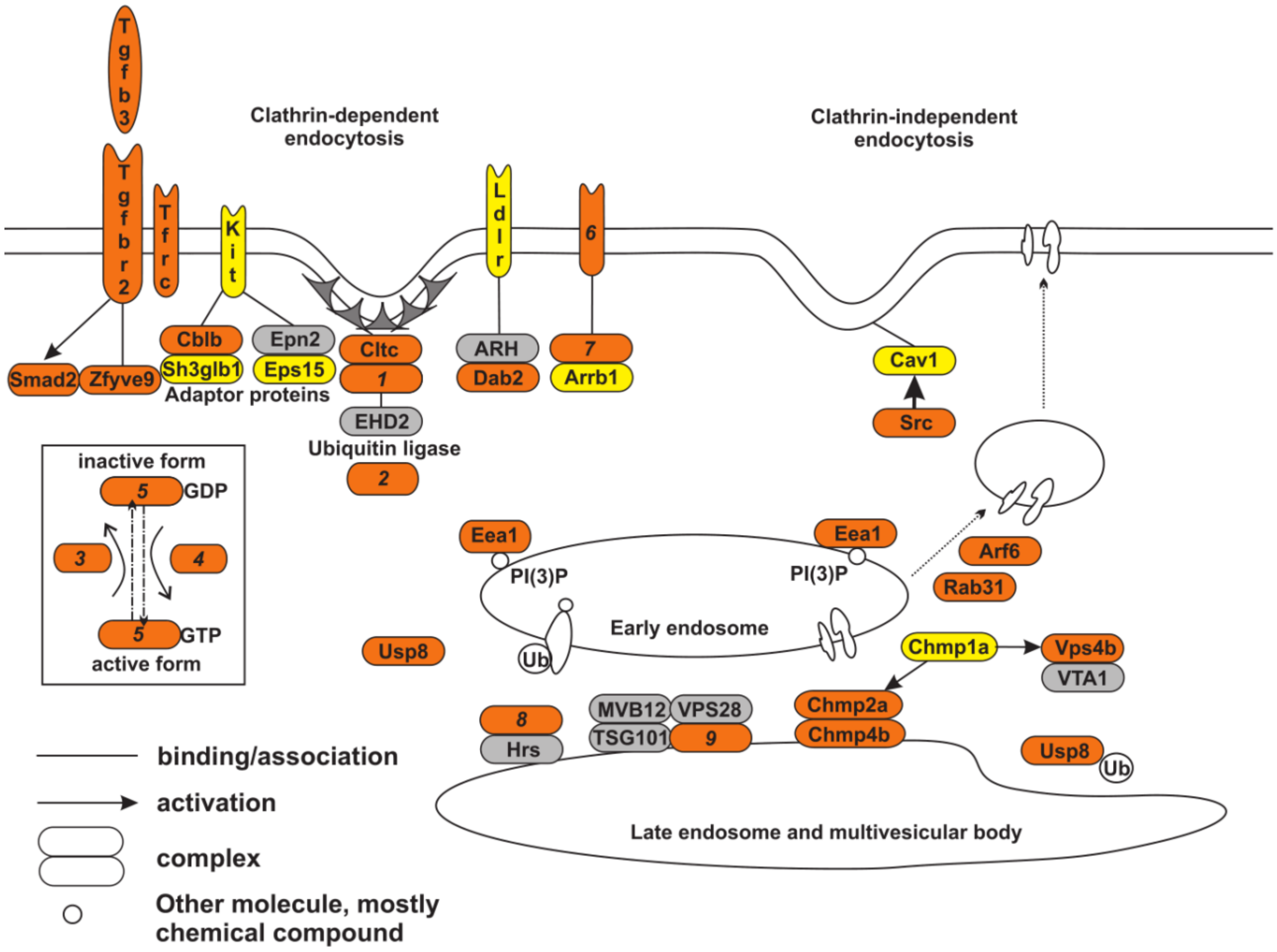

Figure S2: DIANA mirPath analysis predicted that 50 genes involved in the endocytosis pathway are targeted by 3 miRNA from this group. *1*: Ap2b1 and Ap2a2; *2*: Wwp1, Nedd4l, Traf6, Mdm2 and Nedd4; *3*: Asap2, Arfgap3, Agap1, Asap1 and Arap2; *4*: Arfgef1, Arfgef2 and Psd3; *5*: Arf3, Arf6; *6*: F2r and Adrb1; *7*: Grk5, Grk4 and Adrbk1; *8*: Stam and Stam2; *9*: Vps37b and Vps37a.

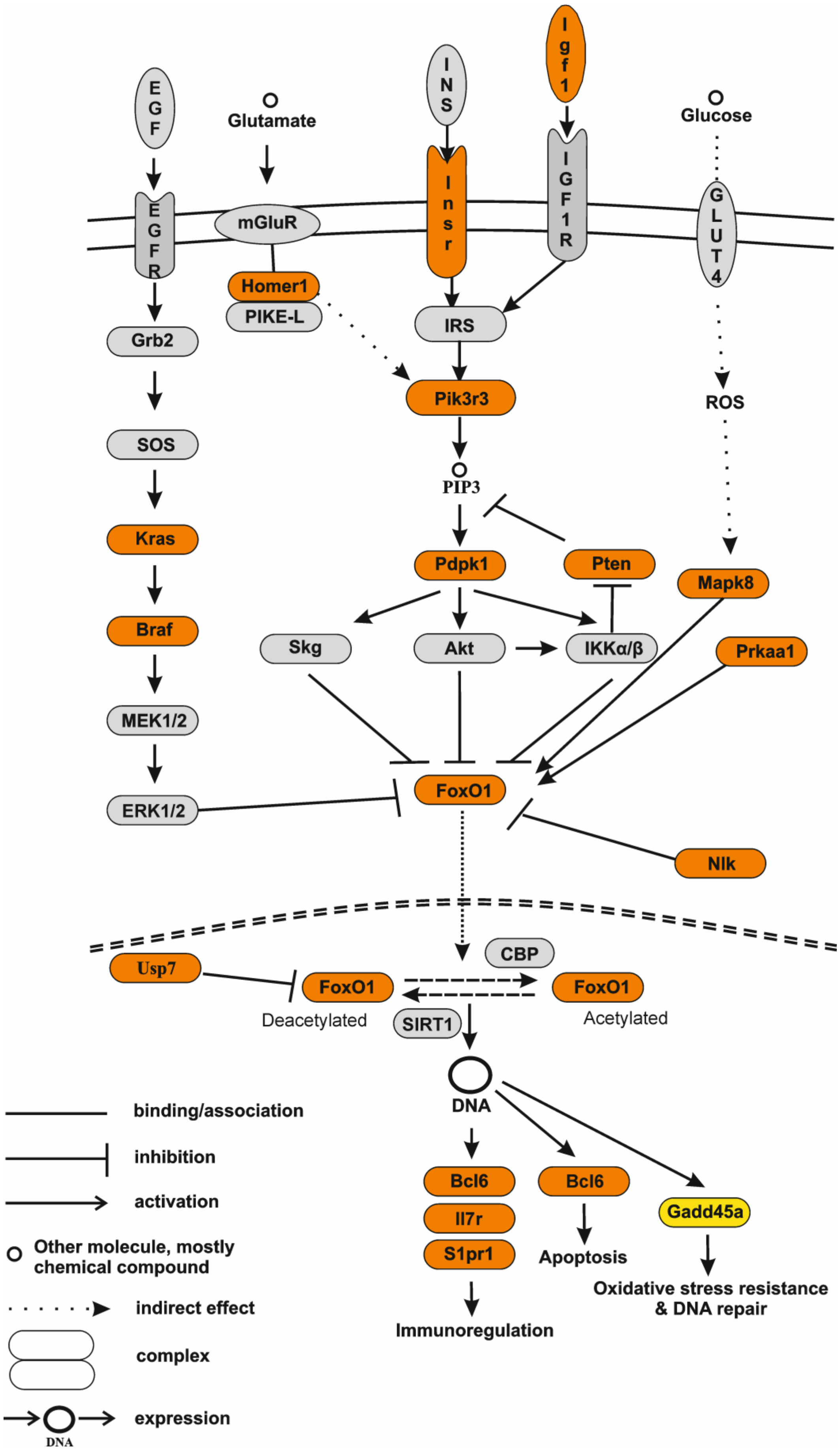

Figure S3: DIANA mirPath analysis predicted that 28 genes involved in the FoxO signalling pathway are targeted by two miRNA from this group.

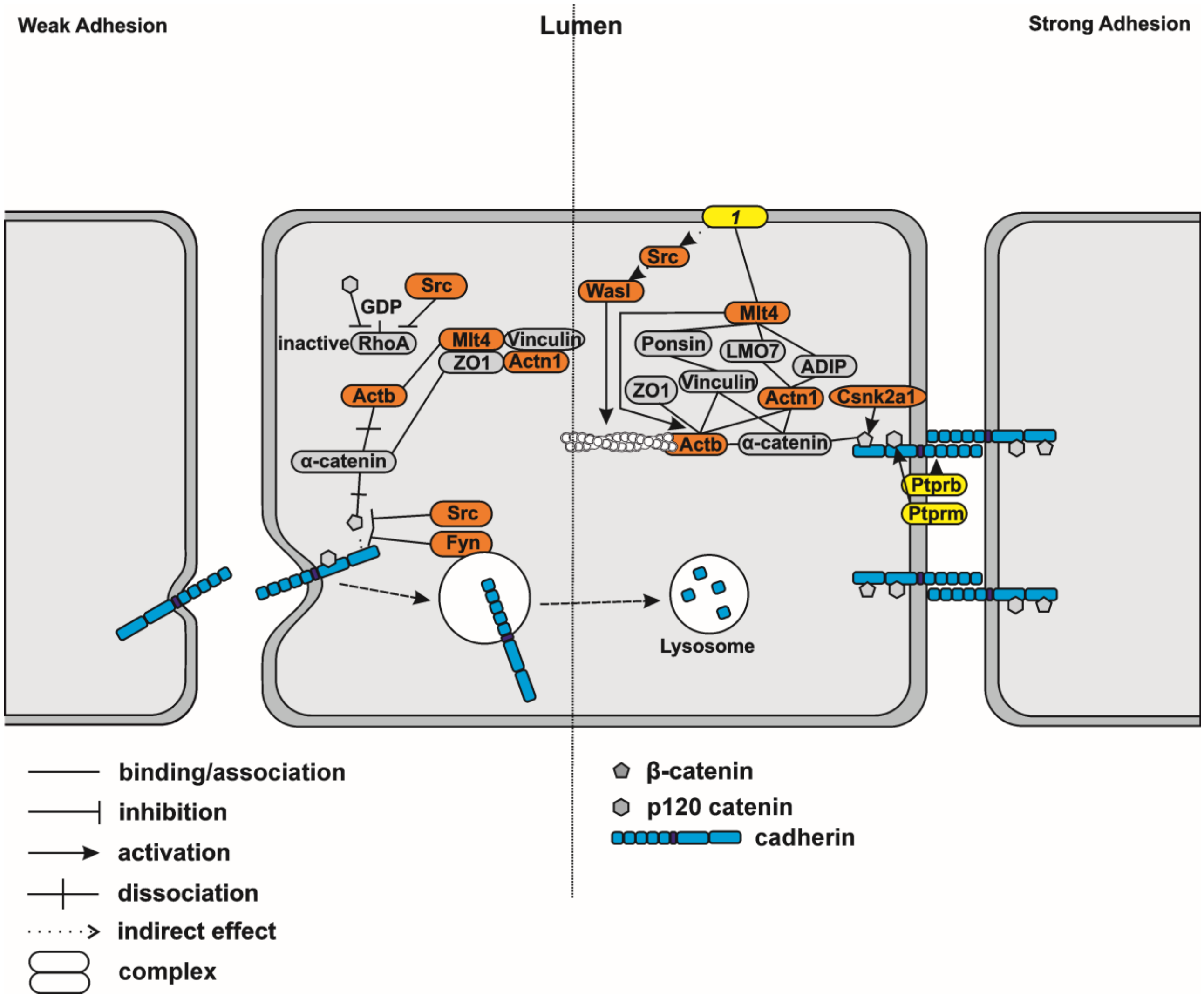

Figure S4: DIANA mirPath analysis predicted that 20 genes involved in the adherens junctions' pathway are targeted by two miRNA from this group. *1*: Pvrl1, Pvrl3.

Table S1. Description of those genes targeted by miRNA upregulated that showed a higher abundance in PbA infected mice in comparison with Py-infected mice

**Pathway**	**Protein**	**miRNA targeting the protein**	**Protein function**
TGF-β signalling pathway	Tgfb3	miR-19a-3p, miR-19b-3p	Binds to the type II TGF-β receptor, which then binds to the type I TGF-β receptor[1]
	Inhbb	miR-19b-3p	Encodes the activin/inhibin βB subunit of activins and inhibins, belonging to the TGF-β super-family[2]
	Tnf	miR-19a-3p, miR-19b-3p	Encodes TNF, which can induce the transcription of I-Smads (Smad6/Smad 7)[3]
	Tgfbr2	miR-19a-3p, miR-19b-3p	Encodes the TGFβRII receptor, which phosphorylates and activates TGFβRI[4]
	Acvr1b	miR-19a-3p, miR-19b-3p	Encodes the Activin A receptor type 1b protein, which is a transmembrane serine/ threonine kinase receptor and is essential for signalling[5]
	Acvr2a	miR-19b-3p	Codes for an Activin receptor type II that is able to bind Activin ligands[6]
	Bmpr2	miR-19a-3p, miR-19b-3p	Encodes the BMP type-II receptor, which appears to bind exclusively to BMP ligands[7]
	Bmpr1a	miR-19a-3p, miR-19b-3p	Encodes the BMP type IA receptor for bone morphogenetic proteins (BMPs)[8]
	Smad2	miR-19a-3p, miR-19b-3p	The intracellular signals for the activins and TGF-βs are mediated through the phosphorylation of Smad2[7]
	Smad4	miR-19a-3p, miR-19b-3p	Forms heterotrimeric complexes with two R-Smads, which are translocated to the nucleus[9]
	Ppp2ca	miR-19a-3p, miR-19b-3p	Encodes the catalytic subunit of a serine phosphatase PP2A, which binds to type I receptors[9]
	Ppp2r1b	miR-19a-3p, miR-19b-3p	Encodes the β isoform of the A subunit of the serine/threonine protein phosphatase 2A (PP2A), which binds to type I receptors[10]
	Zfyve9	miR-19a-3p, miR-19b-3p	Encodes SARA, which functions as a linker protein between type I activin/TGF-β receptors and SMAD2/3 to stimulate SMAD phosphorylation[11]
	Sp1	miR-19a-3p, miR-19b-3p	Cooperates with Smad2, Smad3 and Smad4 to induce $p15^{Ink4B}$ transcription in response to TGF-β[12]
	Id3	miR-19a-3p, miR-19b-3p	Id proteins are important parts of signalling pathways involved in development, cell cycle and tumorigenesis[13]

Endocytosis	Tgfb3	miR-19a-3p, miR-19b-3p	Encodes a member of the TGF-β family of proteins[14]
	Tgfbr2	miR-19a-3p, miR-19b-3p	Encodes a cell-surface receptor for TGF-β[15]
	Tfrc	miR-19a-3p, miR-19b-3p	Encodes a membrane receptor for transferrin[16]
	Kit	miR-19b-3p	Encodes receptor tyrosine kinases, such as the EGFRs[17]
	Smad2	miR-19a-3p, miR-19b-3p	SARA/Smad2 complex allows signalling and recycling[18]
	Zfyve9	miR-19a-3p, miR-19b-3p	Encodes SARA protein, which facilitates signal transduction by promoting the association of SMAD2 and SMAD3 with receptor complexes[19]
	Cblb	miR-19a-3p, miR-19b-3p	Encodes Cbl, which mediates the internalisation of many receptors[17]
	Sh3glb1	miR-19b-3p	Encodes endophilins, which induce membrane curvature and help in the fission of clathrin-coated buds from the membrane[17]
	Eps15	miR-19b-3p	EPS15 and epsin might bind to monoubiquitylated EGFRs to move EGFRs out of lipid rafts[17]
	Cltc	miR-19a-3p, miR-19b-3p	Encodes a major subunit of clathrin, which serves as the vesicle coat protein involved in intracellular trafficking and endocytosis[20]
	Ap2a2	miR-19b-3p	Encodes the adaptor protein-2 (AP2), mediates the endocytosis of cargo proteins[17]
	Ap2b1	miR-19a-3p, miR-19b-3p	Encodes one of the two large chain components of the clathrin assembly protein complex 2, which is important for protein transport[21]
	Wwp1	miR-19a-3p, miR-19b-3p	Encodes a ubiquitin ligase that targets proteins for degradation or other cellular fates[22]
	Traf6	miR-19a-3p, miR-19b-3p	Encodes a ubiquitin ligase that participates in signal transduction of both the TNF receptor superfamily and the interleukin-1 receptor/Toll-like receptor superfamily[23]
	Nedd4	miR-19a-3p, miR-19b-3p	Plays a crucial role in ubiquitination of many plasma membrane proteins[24]
	Nedd4l	miR-19a-3p, miR-19b-3p	Encodes Nedd4-2, which binds and regulates a number of membrane proteins to aid in their internalisation and turnover[25]
	Mdm2	miR-19a-3p, miR-19b-3p	Encodes an E3-ubiquitin ligase that promotes the proteasomal degradation of the cell-cycle regulator p53[26]
	Arf6	miR-19a-3p, miR-19b-3p	Controls the redelivery to the plasma membrane[27]
	Ldlr	miR-19b-3p	Encodes a membrane receptor for low-density lipoprotein (LDL) particles, which constitutes a clathrin-dependent endocytosis cargo protein[28]
	F2r	miR-19a-3p, miR-19b-3p	Encodes G-protein coupled receptors (GPCR) phosphorylated by GPCR kinases (GRKs), allowing β-arrestins to bind to the GPCR[27]

Adrb1	miR-19b-3p	Encodes the β1-adrenergic receptor, a G-protein coupled receptor (GPCR)[29]
Dab2	miR-19a-3p, miR-19b-3p	Encodes the protein Disabled-2 (Dab2), which is capable of direct interaction with LDL receptors[30]
Grk4	miR-19b-3p	Encodes a GPCR kinase (GRK) that phosphorylates GPCR, allowing β-arrestins to bind to the GPCR[27]
Grk5	miR-19a-3p, miR-19b-3p	As above[27]
Adrbk1	miR-19a-3p, miR-19b-3p	As above[31]
Arrb1	miR-19b-3p	Encodes β-arrestin that binds to the GPCR, preventing G-protein recruitment and thereby terminating signalling[27]
Rab11b	miR-19b-3p	Signalling molecule required for fusion back to the plasmatic membrane[32]
Eea1	miR-19a-3p, miR-19b-3p	Encodes the early endosomal antigen 1 (EEA1), recruited to membranes by phosphatidylinositol 3-phosphate and Rab5-GTP, together facilitating early endosome fusion[32]
Usp8	miR-19a-3p, miR-19b-3p	Encodes the UBPY protein, which can deubiquitinate cargos and direct them to different fates[18]
Stam	miR-19a-3p, miR-19b-3p	In early endosomes, STAM forms a ternary complex with HRS and EPS15 that interacts with EGFRs[17]
Stam2	miR-19a-3p, miR-19b-3p	A regulator of receptor signalling and trafficking that interacts directly with Hrs[33]
Eea1	miR-19a-3p, miR-19b-3p	Encodes an effector of the small GTPase Rab5 that controls early-endosome fusion dynamics[17]
Chmp4b	miR-19a-3p, miR-19b-3p	Encodes a member of the family of small coiled-coil proteins named CHMP implicated in playing roles in multivesicular body sorting[34]
Chmp1a	miR-19b-3p	As above[34]
Chmp2a	miR-19a-3p, miR-19b-3p	As above[34]
Vps4b	miR-19a-3p, miR-19b-3p	Encodes a protein responsible for the disassembly of ESCRT-III[35]
Rab31	miR-19a-3p, miR-19b-3p	Encodes Rab22, required for recycling back out of the plasmatic membrane[32]
Arf3	miR-19a-3p, miR-19b-3p	Small GTPase required for the integrity of recycling endosomes and the recycling pathway[36]
Vps37a	miR-19b-3p	Encodes the protein Vps37, which together with Tsg101, Vps28 and Mvb12, forms the ESCRT-I complex[37]
Vps37b	miR-19b-3p	As above[18]
Cav1	miR-19b-3p	Encodes caveolin-1, shown to be important for the formation of caveolae[17]

Src	miR-19a-3p, miR-19b-3p	Its activation leads to plasmatic membrane ruffling and corresponding macropinocytosis[32]
Agap1	miR-19b-3p	Encodes an endosome-associated ARF-GAP protein that affects the actin cytoskeleton[38]
Asap1	miR-19a-3p, miR-19b-3p	Belongs to a family of multifunctional scaffold proteins that regulate membrane trafficking and actin remodelling[39]
Asap2	miR-19a-3p, miR-19b-3p	Encodes an Arf-GAP that has been reported to bind proteins that function as part of the endocytic machinery[40]
Arfgap3	miR-19a-3p, miR-19b-3p	A GTPase-activating protein that associates with the Golgi apparatus and regulates the vesicular trafficking pathway[41]
Arap2	miR-19b-3p	Encodes an ArfGAP implicated in the regulation of actin and cell motility[42]
Arfgef1	miR-19a-3p, miR-19b-3p	Encodes a protein responsible for the activation of Arf GTPases[43]
Arfgef2	miR-19a-3p, miR-19b-3p	Encodes the brefeldin A-inhibited GEF2 protein (BIG2), required for vesicle and membrane trafficking from the trans-Golgi network[44]
Psd3	miR-19a-3p, miR-19b-3p	Encodes a guanine nucleotide exchange factor for the small GTPase ARF6, which regulates membrane trafficking and the actin cytoskeleton[45]

References

1 Xu, Y. & Pasche, B. TGF-beta signaling alterations and susceptibility to colorectal cancer. *Hum Mol Genet* **16 Spec No 1**, R14-20, doi:10.1093/hmg/ddl486 (2007).

2 Kevelam, S. H. *et al.* A patient with a mild holoprosencephaly spectrum phenotype and heterotaxy and a 1.3 Mb deletion encompassing GLI2. *Am J Med Genet. Part A* **158a**, 166-173, doi:10.1002/ajmg.a.34350 (2012).

3 Yan, X., Liu, Z. & Chen, Y. Regulation of TGF-beta signaling by Smad7. *Acta Biochim Biophys Sin* **41**, 263-272 (2009).

4 Flavell, R. A., Sanjabi, S., Wrzesinski, S. H. & Licona-Limon, P. The polarization of immune cells in the tumour environment by TGFbeta. *Nat Rev Immunol* **10**, 554-567, doi:10.1038/nri2808 (2010).

5 Huang, C. W. *et al.* Gene expression of human lung cancer cell line CL1-5 in response to a direct current electric field. *PLoS One* **6**, e25928, doi:10.1371/journal.pone.0025928 (2011).

6 van Dijk, M. & Oudejans, C. (Epi)genetics of pregnancy-associated diseases. *Front Genet* **4**, 180, doi:10.3389/fgene.2013.00180 (2013).

7 Shimasaki, S., Moore, R. K., Otsuka, F. & Erickson, G. F. The bone morphogenetic protein system in mammalian reproduction. *Endocr Rev* **25**, 72-101, doi:10.1210/er.2003-0007 (2004).

8 Miura, S., Davis, S., Klingensmith, J. & Mishina, Y. BMP signaling in the epiblast is required for proper recruitment of the prospective paraxial mesoderm and development of the somites. *Development (Cambridge, England)* **133**, 3767-3775, doi:10.1242/dev.02552 (2006).

9 Derynck, R. & Zhang, Y. E. Smad-dependent and Smad-independent pathways in TGF-beta family signalling. *Nature* **425**, 577-584, doi:10.1038/nature02006 (2003).

10 Wang, S. S. *et al.* Alterations of the PPP2R1B gene in human lung and colon cancer. *Science* **282**, 284-287 (1998).

11 Chang, H., Brown, C. W. & Matzuk, M. M. Genetic analysis of the mammalian transforming growth factor-beta superfamily. *Endocr Rev* **23**, 787-823, doi:10.1210/er.2002-0003 (2002).

12 Feng, X. H., Lin, X. & Derynck, R. Smad2, Smad3 and Smad4 cooperate with Sp1 to induce p15(Ink4B) transcription in response to TGF-beta. *EMBO J***19**, 5178-5193, doi:10.1093/emboj/19.19.5178 (2000).

13 Ruzinova, M. B. & Benezra, R. Id proteins in development, cell cycle and cancer. *Trends Cell Biol* **13**, 410-418 (2003).

14 Kuechler, A. *et al.* Exome sequencing identifies a novel heterozygous TGFB3 mutation in a disorder overlapping with Marfan and Loeys-Dietz syndrome. *Mol Cell Probe* **29**, 330-334, doi:10.1016/j.mcp.2015.07.003 (2015).

15 Bignell, G. R. *et al.* Signatures of mutation and selection in the cancer genome. *Nature* **463**, 893-898, doi:10.1038/nature08768 (2010).

16 Stevens, S. G., Gardner, P. P. & Brown, C. Two covariance models for iron-responsive elements. *RNA Biol* **8**, 792-801, doi:10.4161/rna.8.5.16037 (2011).

17 Le Roy, C. & Wrana, J. L. Clathrin- and non-clathrin-mediated endocytic regulation of cell signalling. *Nat Rev Mol Cell Bio* **6**, 112-126, doi:10.1038/nrm1571 (2005).

18 Acconcia, F., Sigismund, S. & Polo, S. Ubiquitin in trafficking: the network at work. *Exp Cell Res* **315**, 1610-1618, doi:10.1016/j.yexcr.2008.10.014 (2009).

19 Sumegi, J. *et al.* Gene-expression signatures differ between different clinical forms of familial hemophagocytic lymphohistiocytosis. *Blood* **121**, e14-24, doi:10.1182/blood-2012-05-425769 (2013).

20 DeMari, J. *et al.* CLTC as a clinically novel gene associated with multiple malformations and developmental delay. *Am J Med Genet. Part A* **170a**, 958-966, doi:10.1002/ajmg.a.37506 (2016).
21 Schmid, E. M. *et al.* Role of the AP2 beta-appendage hub in recruiting partners for clathrin-coated vesicle assembly. *PLoS Biol* **4**, e262, doi:10.1371/journal.pbio.0040262 (2006).
22 Rotin, D. & Kumar, S. Physiological functions of the HECT family of ubiquitin ligases. *Nat Rev Mol Cell Bio* **10**, 398-409, doi:10.1038/nrm2690 (2009).
23 Ye, H. *et al.* Distinct molecular mechanism for initiating TRAF6 signalling. *Nature* **418**, 443-447, doi:10.1038/nature00888 (2002).
24 Rotin, D., Staub, O. & Haguenauer-Tsapis, R. Ubiquitination and endocytosis of plasma membrane proteins: role of Nedd4/Rsp5p family of ubiquitin-protein ligases. *J Membrane Biol* **176**, 1-17 (2000).
25 Goel, P., Manning, J. A. & Kumar, S. NEDD4-2 (NEDD4L): the ubiquitin ligase for multiple membrane proteins. *Gene* **557**, 1-10, doi:10.1016/j.gene.2014.11.051 (2015).
26 Bonifacino, J. S. & Traub, L. M. Signals for sorting of transmembrane proteins to endosomes and lysosomes. *Annu Rev Biochem* **72**, 395-447, doi:10.1146/annurev.biochem.72.121801.161800 (2003).
27 Scita, G. & Di Fiore, P. P. The endocytic matrix. *Nature* **463**, 464-473, doi:10.1038/nature08910 (2010).
28 Grant, B. D. & Donaldson, J. G. Pathways and mechanisms of endocytic recycling. *Nat Rev Mol Cell Biol* **10**, 597-608, doi:10.1038/nrm2755 (2009).
29 Foster, S. R. *et al.* Expression, regulation and putative nutrient-sensing function of taste GPCRs in the heart. *PLoS One* **8**, e64579, doi:10.1371/journal.pone.0064579 (2013).
30 Sorkin, A. Cargo recognition during clathrin-mediated endocytosis: a team effort. *Curr Opin Cell Biol* **16**, 392-399, doi:10.1016/j.ceb.2004.06.001 (2004).
31 Carman, C. V., Lisanti, M. P. & Benovic, J. L. Regulation of G protein-coupled receptor kinases by caveolin. *J Biol Chem* **274**, 8858-8864 (1999).
32 Donaldson, J. G., Porat-Shliom, N. & Cohen, L. A. Clathrin-independent endocytosis: a unique platform for cell signaling and PM remodeling. *Cell Signal* **21**, 1-6, doi:10.1016/j.cellsig.2008.06.020 (2009).
33 Bache, K. G., Raiborg, C., Mehlum, A. & Stenmark, H. STAM and Hrs are subunits of a multivalent ubiquitin-binding complex on early endosomes. *J Biol Chem* **278**, 12513-12521, doi:10.1074/jbc.M210843200 (2003).
34 Katoh, K., Shibata, H., Hatta, K. & Maki, M. CHMP4b is a major binding partner of the ALG-2-interacting protein Alix among the three CHMP4 isoforms. *Arch Biochem Biophys* **421**, 159-165 (2004).
35 Lata, S. *et al.* Helical structures of ESCRT-III are disassembled by VPS4. *Science* **321**, 1354-1357, doi:10.1126/science.1161070 (2008).
36 Kondo, Y. *et al.* ARF1 and ARF3 are required for the integrity of recycling endosomes and the recycling pathway. *Cell Struct Funct* **37**, 141-154 (2012).
37 Raiborg, C. & Stenmark, H. The ESCRT machinery in endosomal sorting of ubiquitylated membrane proteins. *Nature* **458**, 445-452, doi:10.1038/nature07961 (2009).
38 Nie, Z. *et al.* AGAP1, an endosome-associated, phosphoinositide-dependent ADP-ribosylation factor GTPase-activating protein that affects actin cytoskeleton. *J Biol Chem* **277**, 48965-48975, doi:10.1074/jbc.M202969200 (2002).
39 Randazzo, P. A. & Hirsch, D. S. Arf GAPs: multifunctional proteins that regulate membrane traffic and actin remodelling. *Cell Signal* **16**, 401-413 (2004).

40 Inoue, H. & Randazzo, P. A. Arf GAPs and their interacting proteins. *Traffic (Copenhagen, Denmark)* **8**, 1465-1475, doi:10.1111/j.1600-0854.2007.00624.x (2007).
41 Obinata, D. *et al.* ARFGAP3, an androgen target gene, promotes prostate cancer cell proliferation and migration. *Int J Cancer* **130**, 2240-2248, doi:10.1002/ijc.26224 (2012).
42 Yoon, H. Y. *et al.* ARAP2 effects on the actin cytoskeleton are dependent on Arf6-specific GTPase-activating-protein activity and binding to RhoA-GTP. *J Cell Sci* **119**, 4650-4666, doi:10.1242/jcs.03237 (2006).
43 Aizel, K. *et al.* Integrated conformational and lipid-sensing regulation of endosomal ArfGEF BRAG2. *PLoS Biol* **11**, e1001652, doi:10.1371/journal.pbio.1001652 (2013).
44 Sheen, V. L. *et al.* Mutations in ARFGEF2 implicate vesicle trafficking in neural progenitor proliferation and migration in the human cerebral cortex. *Nat Genet* **36**, 69-76, doi:10.1038/ng1276 (2004).
45 Sakagami, H. *et al.* Distinct spatiotemporal expression of EFA6D, a guanine nucleotide exchange factor for ARF6, among the EFA6 family in mouse brain. *Brain Res* **1093**, 1-11, doi:10.1016/j.brainres.2006.02.058 (2006).
